# Perls' Prussian blue staining and chemistry of Prussian blue and Turnbull blue

**DOI:** 10.1016/j.fsisyn.2025.100627

**Published:** 2025-07-10

**Authors:** Ai Sonoda, Masayuki Nihei, Norihiro Shinkawa, Eiji Kakizaki, Nobuhiro Yukawa

**Affiliations:** a Division of Legal Medicine, Department of Social Medicine, Faculty of Medicine, University of Miyazaki, Japan; b Department of Chemistry, Institute of Pure and Applied Sciences, University of Tsukuba, Japan

**Keywords:** Perls' reaction, Skin wounds, Bruise, Haemosiderin-laden macrophages, Glycophorin A, Forensic pathology

## Abstract

Perls’ Prussian blue staining or reaction is used to detect haemosiderin, which is stored or sequestrated non-haeme iron. Methodologically, the iron in haemosiderin is released as Fe^3+^ (Fe^III^) by hydrochloric acid (HCl), and Fe^III^ reacts with potassium ferrocyanide (K_4_[Fe^II^(CN)_6_]) to form Prussian blue. Iron released from ferritin, another stored non-haeme iron, is too scarce to be detected. Haeme iron, including haemoglobin and cytochromes, is not released by HCl. Thus, haemosiderin can easily be detected under the microscope as distinct blue deposits with minimal background staining. The chemistry of cyanide-bridged iron complexes, including Prussian blue and Turnbull blue, is the basis for understanding Perls' staining. Prussian blue is a cubic lattice with Fe^II^ or Fe^III^ ions alternately aligned at the corners to give Fe^II^–CN–Fe^III^ formations at the edges. Physicochemically, Prussian blue is soluble in water (dispersible as a colloid) or insoluble depending on how it is formed. As with Perls' staining, Prussian blue is expected to take a soluble form because of excess K_4_[Fe^II^(CN)_6_] compared to Fe^3+^ released from tissues. Notably, Prussian blue used in Perls' staining does not in fact dissipate into the staining solution but remains on the tissue, rendering this a practical method for histological detection of haemosiderin and also exogenous iron of forensic significance. However, further examinations of its mechanisms are needed to evaluate the applicability of this method on various forensics cases.

## 1 Introduction

Perls' Prussian blue staining is used to microscopically detect haemosiderin to estimate time since bleeding [1,2]. However, the current understanding of Perls' staining is often insufficient. In this review, we briefly introduce the history and characteristics of Perls' staining and focus on the structural chemistry of Prussian blue, haemosiderin as a stainable non-haeme iron, redox chemistry of Prussian blue and Turnbull blue, which is the basic mechanism of Perls’ staining. Finally, we briefly introduce and discuss staining solutions and the detection of haemosiderin-laden macrophages for dating skin wounds.

## 2 History of Prussian blue

### 2.1 Discovery

The discovery of Prussian blue and a reproducible production method by chemist Johann Jacob von Diesbach in the laboratory of alchemist Johann Konrad Dippel in 1706 in Berlin was described in an article in German [3], which was translated into English [3]. By calcinating animal blood in the presence of potassium carbonate (potash), the nitrogen in the blood was transferred to potash as thermally stable cyanic acid (HCN) to yield potassium cyanide (KCN). By mixing the leachate of calcined blood consisting of potash and KCN with iron-containing alum, cyanide was transferred to iron to yield a potassium ferrocyanide (potassium hexacyanoferrate (II), K_4_[Fe^II^(CN)_6_]) complex, which reacts with iron to produce a blue substance (Prussian blue).

### 2.2 Worldwide reach

Prussian blue is widespread worldwide as a reasonably priced and magnificent blue paint and has impacted cultural development in the century since its discovery. In Japan, Katsushika Hokusai used Prussian blue to effectively express the depth of the sea in the print "Under the Wave off Kanagawa" (1830) [3], which became an inspiration for the composer Claude Debussy.

Regarding medical usage, Grohé [4] found in a study on melanaemia that black pigments that form in greenish tissues of putrefied bodies were not melanin but iron compounds, based on the pigments turning to Prussian blue upon the addition of K_4_[Fe^II^(CN)_6_] and HCl. Based on this finding, Max Perls [5] in 1867 applied Prussian blue and Turnbull blue to systematically detect iron in various tissues and concluded that Prussian blue was suitable for this purpose because tissue iron was present in the ferric form. A representative intensely stainable tissue is the inner membrane in internal haemorrhagic pachymeningitis.

## 3 Structural chemistry of Prussian blue

### 3.1 Lattices of small cubes

**Fig. 1:**
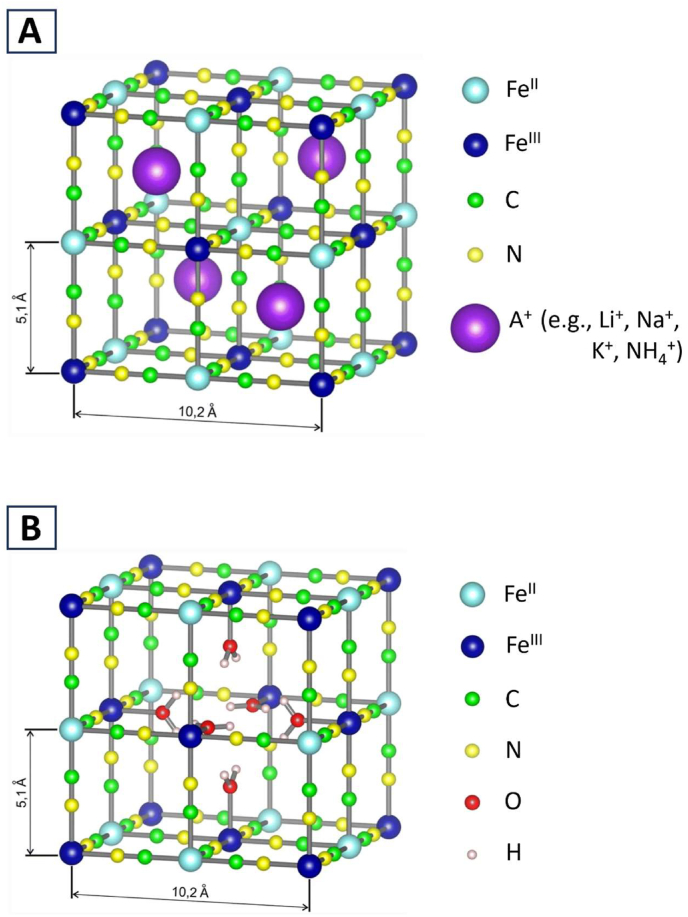
Conventional view of the lattice structure of soluble and insoluble Prussian blue (reproduced from Kraft [7] with permission from the publisher). In both soluble and insoluble Prussian blue, the lattice consists of small cubes with Fe^II^ (light blue) or Fe^III^ (dark blue) at the corners and Fe^II^–C–N–Fe^III^ at the edges. (A) Soluble Prussian blue. The structure is characterized by a complete lattice with a 1:1 ratio of Fe^III^:Fe^II^. In other words, the ratio of Fe^3+^:[Fe^II^(CN)_4_]^4–^ is 1:1, and alkali ions (A^+^) are incorporated in alternate octans to balance the electrical charge. (B) Insoluble Prussian blue. The structure is characterized by vacancies of [Fe^II^(CN)_4_]^4–^ with a 4:3 ratio of Fe^III^:Fe^II^. In other words, the ratio of Fe^3+^:[Fe^II^(CN)_4_]^4–^ is 4:3, rendering the structure electrically neutral without alkali ions. The vacant space is surrounded by water molecules (H_2_O, red and two yellow small spheres) that coordinate to Fe^3+^.

Prussian blue is formed by the reaction of ferric ions (Fe^3+^) hexahydrated in aqueous solution as (Fe^III^(H_2_O)_6_^3+^) with K_4_[Fe^II^(CN)_6_] [6]. The resulting structure is a cubic lattice composed of iron ions with alternating divalent (Fe^II^) and trivalent (Fe^III^) ions at the corners of small cubes. The cyanide ligands (–CN–) bridge Fe^II^ and Fe^III^ such that the C and N atoms of the cyanide ligand coordinate with the Fe^II^ and Fe^III^ ions, respectively, forming (Fe^II^–C–N–Fe^III^) [6,7], as shown in Fig. 1.

### 3.2 Soluble and insoluble Prussian blue

Physicochemically, Prussian blue may be soluble (strictly speaking, dispersed as a colloid) or insoluble in water, depending primarily on the reaction conditions under which it is formed [6,7]. A typical condition under which soluble Prussian blue is produced is the reaction of Fe^3+^ with K_4_[Fe^II^(CN)_6_] in excess, whereas the reaction of K_4_[Fe^II^(CN)_6_] with Fe^3+^ in excess produces an insoluble form. Traditionally, the composition of soluble Prussian blue has been described as KFe^III^[Fe^II^(CN)_6_] molecules with a 1:1 ratio of Fe^III^: Fe^II^ along with potassium ions (K^+^) enclosed in small cubes that balance the electrical charge of the molecule (Fig. 1A) [6,7]. In contrast, insoluble Prussian blue is described as Fe^III^_4_[Fe^II^(CN)_6_]_3_ with a 4:3 ratio of Fe^III^: Fe^II^ and having ferrocyanide vacancies (Fig. 1B).

**Fig. 2:**
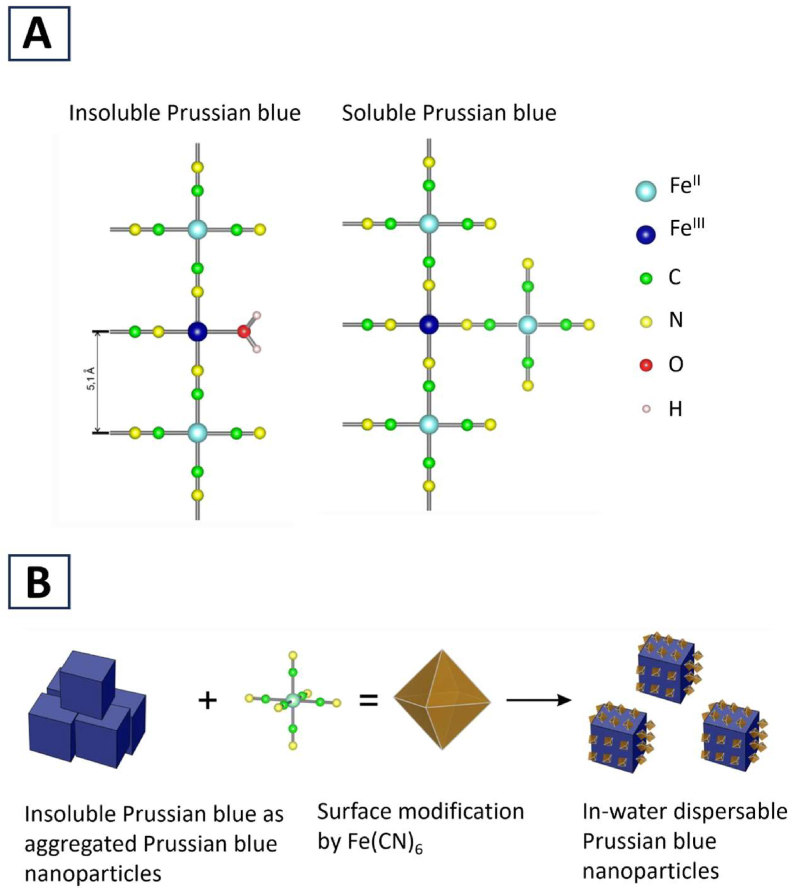
Current view of the difference between insoluble and soluble Prussian blue (reproduced from Kraft [7] with permission from the publisher). (A) Small surface cut-out of Prussian blue. In either the insoluble or soluble form, the left side is within the lattice and the right side is outside. The insoluble form is characterized by a water molecule that coordinates to surface Fe^III^. The soluble form is characterized by the replacement of the water molecule with an additional [Fe^II^(CN)_4_]^4–^. (B) Schematic of experiment supporting the current view. The insoluble form (aggregated cubes, left) suspended in water can be converted to its soluble form (dispersed cubes with modified surfaces, right) by adding [Fe^II^(CN)_4_]^4–^ (ball-and-stick model and its regular octahedral structure (brown)).

Industrial methods for producing insoluble Prussian blue with few ferrocyanide vacancies have been developed by controlling the precipitation rate. Consequently, it was not the presence of vacancies but rapid precipitation that was found to render Prussian blue insoluble [7]. Currently, solubility is postulated to be determined by differences in the outer surface structure. For the insoluble form, a water molecule (H_2_O) is coordinated to each Fe^III^ ion of the outer surface, whereas an additional [Fe^II^(CN)_6_]^4−^ molecule coordinates to each Fe^III^ ion for the soluble form (Fig. 2A) [7]. Insoluble Prussian blue can be converted to its soluble form by suspending it in water, adding K_4_[Fe^II^(CN)_6_], and stirring (Fig. 2B) [7].

## 4 Perls' Prussian blue staining (Perls’ staining)

### 4.1 Perls' original method

Perls assumed that iron in tissues, such as in the dura mater of chronic subdural haematoma and in the skin of aged bruises, exists predominantly as ferric ions (Fe^III^) and iron oxides [5]. He soaked these tissues in K_4_[Fe^II^(CN)_6_] aqueous solution and observed them under a microscope, but the blue colour either did not develop or was very faint. However, the subsequent addition of concentrated hydrochloric acid (HCl) resulted in the development of an intense blue colour, indicating that Fe^III^ was released from the tissue by the acid and reacted with K_4_[Fe^II^(CN)_6_] to form Prussian blue.

### 4.2 Iron released from tissues by acid is mostly trivalent

Divalent iron (Fe^II^) can be detected following its release with HCl and the reaction with K_3_[Fe^III^(CN)_6_] forms a blue substance called Turnbull blue. Both Fe^II^ and Fe^III^ can simultaneously be detected as Turnbull blue by converting Fe^III^ to Fe^II^ with ammonium sulphide ((NH_4_)_2_S), followed by a reaction with K_3_[Fe^III^(CN)_6_] [8,9]. However, researchers have confirmed the hypothesis that most of the iron in tissues is present as Fe^III^ and concluded that the staining method devised by Perls was the most useful [8].

In putrefied tissues [10] or within masses of haemorrhages [11], some Fe^III^ in haemosiderin may be converted into Fe^II^ under reducing conditions. For such samples, performing both Turnbull blue and Perls' staining reveals the presence of Fe^II^ and Fe^III^, respectively. However, from a practical viewpoint, Perls’ staining alone is sufficient to detect haemosiderin because the majority of iron remains as Fe^III^.

## 5 Haemosiderin as a marker for the estimation of time since bleeding

In some tissues and organs, particularly those that engage in the metabolism of haemoglobin and red blood cells, such as the liver, spleen, and bone marrow, haemosiderin may be present in its normal composition [12,13]. In such tissues and organs, the apparent increase of haemosiderin means that metabolic disorders cause iron overload [14].

In other tissues and organs where haemosiderin is not usually present, or in traumatized areas, its presence is of forensic significance [1,2,15]. Most commonly, bruising (blunt cutaneous injuries) is examined in practice, and the detection of haemosiderin-laden macrophages (siderophages) was postulated to mean that three days or more had passed since the bleeding event [16]. This implies that at least three days are required to complete the pathway—macrophages migrate to the site of bleeding and phagocytose red blood cells and/or haemoglobin and haematin that were liberated from haemolyzed blood, then phagocytosed red blood cells, haemoglobin and/or haematin are degraded, and then the liberated free iron, through ferritin as an intermediate, accumulates as haemosiderin in sufficient amounts within the cell to be detectable by Perls' staining [15,17,18].

## 6 Iron species unstainable or stainable by Perls' staining

### 6.1 Unstainable haeme iron

Overall, iron in the body can be categorized into haeme and non-haeme iron [9]. The iron in haeme is strongly coordinated to the four nitrogen atoms of the porphyrin ring. Although the iron in haeme can be released by strong oxidants, it is not released by HCl and is therefore unstainable by Perls’ staining [9,19].

### 6.2 Only stainable non-haeme iron species; haemosiderin

Non-haeme iron species are heterogeneous molecules that include iron species characterized in one of these ways: loosely bound to various low molecular weight organic bases (nonprotein-bound irons), bound to transport proteins, contributing to the structure of proteins such as iron-sulphur proteins and non-haeme iron enzymes, or having a storage or sequestration function (ferritin and haemosiderin) [9].

Similar to any other analytical method, Perls' staining has an inherent limit of detection [20]. If the density of iron is low, the resultant Prussian blue is minute and imperceptible. Thus, iron needs to be present at a high enough density in order to be detected, and iron in haemosiderin meets this criteria.

The existence of haemosiderin was postulated by Neumann in 1888 [21]. At that time the yellow pigment produced by the decomposition of blood was known as "Hämatoidin" (haematoidin, crystalized bilirubin, or crystals mainly composed of bilirubin, which is an end product of the degradation of the porphyrin ring of haeme). Neuman considered that the complex stained blue by Perls’ staining was unlikely to be a simple hydrated iron oxide but rather a complex form of iron also derived from blood and thus coined the term "Hämosiderin.”

### 6.3 Ferritin and haemosiderin

Ferritin is a protein that encloses iron (ferrihydrite phosphate) within a spherical shell. The enclosed iron is generally regarded as being undetectable by Perls’ staining, perhaps because pores opening to external spaces are so small (narrowest diameter is less than that of fully hydrated ferric iron, Fe^III^(H_2_O)_6_^3+^) [22], such that only a small amount of iron would be released by HCl and the resultant Prussian blue would not be detectable by microscopic observation [9]. To observe such small amounts of Prussian blue would require detecting the peroxidase-like activity of Prussian blue with 3,3ʹ-diaminobenzidine tetrahydrochloride (DAB) as the hydrogen donor [9,23].

Haemosiderin has been assumed to represent the iron core of ferritin exposed by the digestion of the spherical protein shell [24,25]. As with species of iron in such a core, they are presumed to be oxo species such as 5Fe^III^_2_O_3_·9H_2_O (ferrihydrite), Fe^III^_2_O_3_ (hematite) and Fe^III^_2_Fe^II^O_4_ (magnetite) [9]. These observations (exposed core and constituent iron being oxo species) taken together explain the fact that iron in haemosiderin is easily released by HCl [9].

## 7 Macroscopic application

### 7.1 Iron distribution in the brain

Perls' staining is recognized as a histological method in forensic pathology. However, in neurological science, it has long been used to macroscopically examine the distribution of iron in the brain [26]. By simply immersing slices of fixed or unfixed brain in Perls' staining solution, some regions such as the globus pallidus, deeper layers of cortical grey matter, substantia nigra, and dentate nucleus were lightly stained blue. The stained iron is mainly ferritin, which cannot be detected microscopically unless enhanced by DAB. Such regions in a cerebral hemisphere were found to be stained green by sodium hydrosulphide (NaSH), suggesting that the green colouration of the brain that is observed in putrefied bodies may primarily be due to the formation of a non-haeme iron-sulphur complex(es) by hydrogen sulphide (H_2_S) [27] rather than that of sulfhaemoglobin [28].

### 7.2 Haemosiderin that looks orange-yellow

**Fig. 3:**
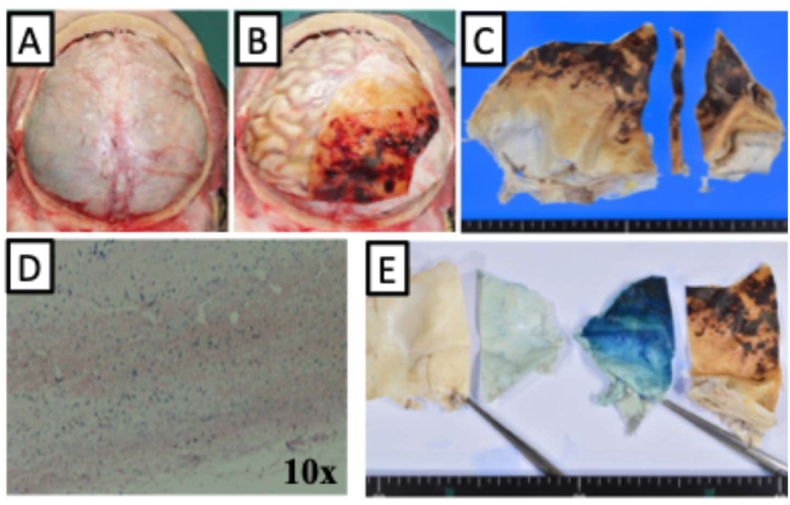
Photograph of an old subdural haemorrhage incidentally found during autopsy. (A) Outer surface of the dura mater. The top is the front. (B) Subdural haemorrhage revealed by the reflection of the left half of the dura. The haemorrhage was thin and appeared old based on its orange-yellow appearance. (C) Inner surface of the formalin-fixed left half of the dura. Strip was stained by Perls' staining. (D) Microscopic image (10× objective) of Perls' staining. The inner surface of the dura is shown at the bottom of the figure. Many blue spots were observed, and at higher magnification, all were identified as haemosiderin-laden macrophages. (F) Immersion of the dura in the staining solution; from left to right, non-immersed and immersed portions of the normal right half of the dura, and immersed and non-immersed portions of the subdural haemorrhage of the left half. All orange-yellow areas were stained blue, indicating that the orange-yellow appearance was indeed attributable to haemosiderin.

Fig. 3A and B shows a thin, old subdural haemorrhage that was incidentally found in a patient who had been on peritoneal dialysis for chronic kidney disease and suddenly died of rupture of an idiopathic haematoma of the sigmoid colon [29]. The haemorrhage appeared old based on its orange-yellow appearance, and Fig. 3C shows the dura after formalin fixation. Microscopically, Perls' staining of a portion (strip shown in Fig. 3C) showed numerous haemosiderin-laden macrophages (Fig. 3D).

Immersion of a different portion of the dura in Perls' staining solution resulted in relatively even blue staining of the inner surface (Fig. 3E). This indicates that the orange-yellow appearance was indeed attributable to haemosiderin. In addition, the relatively even blue staining suggests that haemosiderin was present not only within macrophages but also diffusely in the inner surface and interstitial tissues or spaces. However, as shown in Fig. 3B, only macrophages were observed to be blue under the microscope. Thus, like ferritin distributed in the brain, macroscopic Perls' staining seems to be required for the diffuse distribution of haemosiderin in the tissues to be visualized.

## 8 Redox chemistry of Prussian blue and Turnbull blue

### 8.1 Basic chemistry

The reactions between all possible combinations of Fe^2+^ (Fe^II^), Fe^3+^ (Fe^III^), [Fe^II^(CN)_6_]^4−^, and [Fe^III^(CN)_6_]^3−^ can be calculated as 2 × 2 = 4 [6,30]. The combination of divalent species (Fe^II^ and [Fe^II^(CN)_6_]^4−^) gives Prussian white. Unless under strict anaerobic conditions, Prussian white does not remain white but spontaneously turns to Prussian blue through oxidation by molecular oxygen (O_2_). The conversion to Prussian blue can be rapidly completed with hydrogen peroxide (H_2_O_2_), which acts as an oxidation reagent (H_2_O_2_ + 2H^+^ + 2e^−^→ 2H_2_O). The combination of trivalent species (Fe^III^ and [Fe^III^(CN)_6_]^3−^) results in Prussian brown, a very strong oxidation reagent; if Prussian brown is mixed with H_2_O_2_, H_2_O_2_ acts as a reduction reagent (H_2_O_2_ → O_2_ + 2H^+^ + 2e^−^) to form Prussian blue.

There are two possible combinations of divalent and trivalent species. One is the combination of Fe^III^ and [Fe^II^(CN)_6_]^4−^, which produces Prussian blue, as described above:

(1) Fe^III^_(aq)_ + [Fe^III^(CN)_6_]^4−^_(aq)_ → Fe^III^[Fe^II^(CN)_6_]^−^_(s)_

Where “aq” represents an aqueous solution and “s” represents a solid [6]. In contrast, the combination of Fe^II^ and [Fe^III^(CN)_6_]^3−^ gives Turnbull blue. The structure of Turnbull blue is not ferrous ferricyanide but ferric ferrocyanide, which is the same as Prussian blue [6,29]:

(2) Fe^II^_(aq)_ + [Fe^III^(CN)_6_]^3−^_(aq)_ → Fe^III^[Fe^II^(CN)_6_]^−^_(s)_

### 8.2 Standard electrode potential of Fe^III^/Fe^II^ and [Fe^III^(CN)_6_]^3−^/[Fe^II^(CN)_6_]^4−^ couples

Eqs. (1), (2)) might appear counterintuitive if we consider the following redox potentials:

(3) Fe^III^ + e^−^ → Fe^II^*E*^0^ = 0. 77 V [[31], [32]]

(4) [Fe^III^(CN)_6_]^3−^ + e^−^ → [Fe^II^(CN)_6_]^4−^*E*^0^ = 0. 36 V [32]

Considering the higher reduction potential of the Fe^III^ ion, electrons should transfer from [Fe^II^(CN)_6_]^4−^ to Fe^III^, and thereby Fe^III^ should change to Fe^II^, while [Fe^II^(CN)_6_]^4−^ changes to [Fe^III^(CN)_6_]^3−^. Thus, we may expect that mixing Fe^III^ and [Fe^II^(CN)_6_]^4−^ results in the formation of ferrous ferricyanide Fe^II^[Fe^III^(CN)_6_]^−^. Moreover, mixing Fe^II^ and [Fe^III^(CN)_6_]^3−^ would result in them simply binding to give the same Fe^II^[Fe^III^(CN)_6_]^−^. However, the reactions that actually proceed then result in ferric ferrocyanide Fe^III^[Fe^II^(CN)_6_]^−^, as described by Eqs. (1), (2)).

### 8.3 Outer- or inner-sphere type electron transfer

Generally, electron transfer (redox) reactions between an electron acceptor (A) and electron donor (D) are classified into outer-sphere and inner-sphere types [33]. Outer-sphere types are expressed as A + D → A^−^ + D^+^ (electron transfer not through chemical bonds), whereas the inner-sphere type is expressed as A + D → A−D → A^−^−D^+^ (electron transfer through chemical bonds).

When Fe^III^ and [Fe^II^(CN)_6_]^4−^ are mixed, outer-sphere electron transfer (Fe^III^_(aq)_ + [Fe^II^(CN)_6_]^4−^_(aq)_ → Fe^II^_(aq)_ + [Fe^III^(CN)_6_]^3−^_(aq__)_), which appears to be favourable in terms of redox potential, does not actually occur, and they instead bind to form Fe^III^[Fe^II^(CN)_6_]^−^_(s)_ (Prussian blue).

Similarly, when Fe^II^_(aq)_ and [Fe^III^(CN)_6_]^3−^_(aq)_ are mixed, they bind to form Fe^II^[Fe^III^(CN)_6_]^−^_(s)_. However, inner-sphere type electron transfer from Fe^II^ to Fe^III^ occurs immediately, and Fe^II^[Fe^III^(CN)_6_]^−^ is changed to Fe^III^[Fe^II^(CN)_6_]^−^_(s)_ (Turnbull blue), which is the same as Prussian blue. The mechanisms of each reaction are described in the following sections.

### 8.4 Prussian blue

**Fig. 4:**
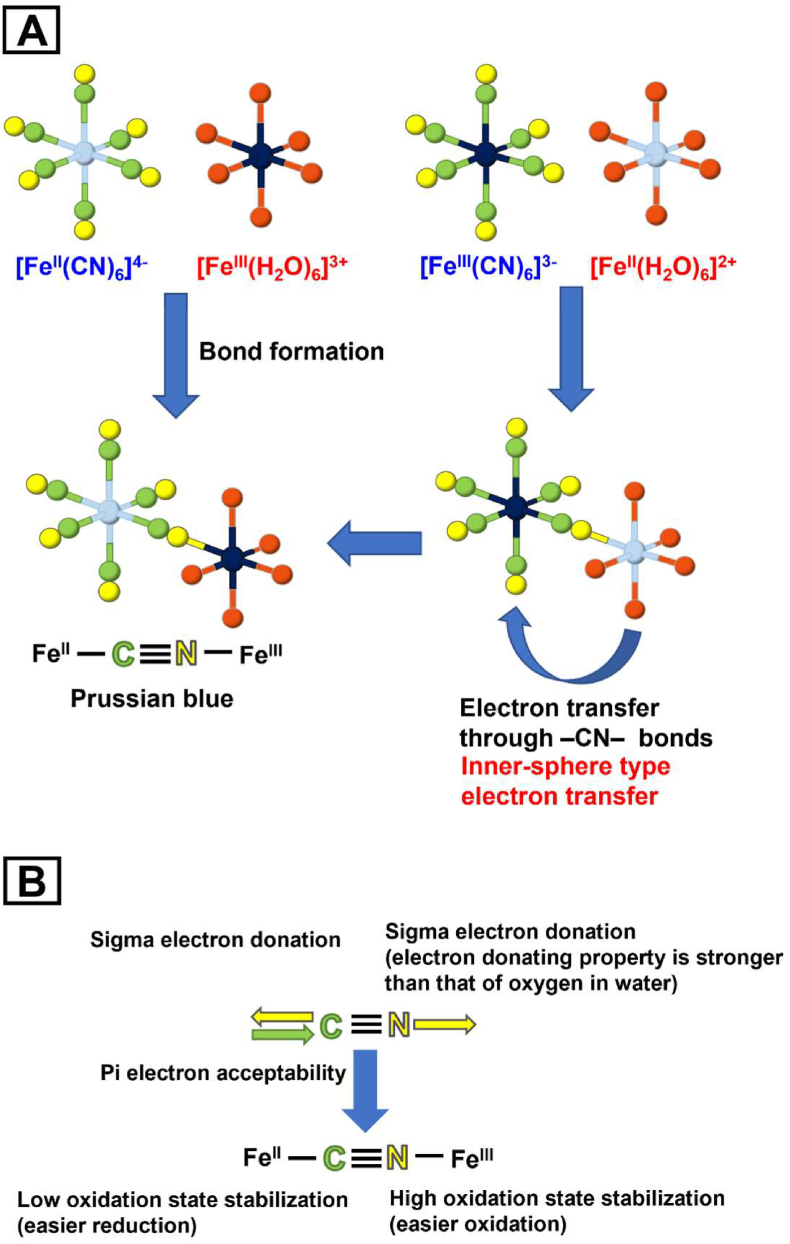
(A) Formation of Prussian blue and Turnbull blue. The structure of Turnbull blue is the same as that of Prussian blue. In the hexacyanoferrate (II) or (III) ions, six cyanide groups are coordinated along the *x*-, *y*-, and *z-axes* ([Fe^II^(CN)_6_]^4-^ or [Fe ^III^(CN)_6_]^3-^). Similarly, iron ions are coordinated by six water molecules in an aqueous solution ([Fe^II^(H_2_O)_6_]^2+^ or [Fe^III^(H_2_O)_6_]^3+^). The colour assignments in the schematics follow that of Fig. 1; Fe^II^ (light blue), Fe^III^ (dark blue), C (green), N (yellow) and O (red). (B) Asymmetric crosslinking ability of cyanide groups. The C atom (green) exhibits both sigma electron-donating and pi electron-accepting characteristics (double arrow), whereas the N atom (yellow) has strong sigma electron-donating power (arrow). This stabilizes a lower oxidation state of Fe^II^ bound to C and a higher oxidation state of Fe^III^ bound to N.

In solution, iron ions exist as hydrates [Fe^II^(H_2_O)_6_]^2+^ or [Fe^III^(H_2_O)_6_]^3+^ in which six water molecules coordinate with the iron ion [[34], [35], [36]]. In the formation of Prussian blue by the reaction of [Fe^III^(H_2_O)_6_]^3+^ and [Fe^II^(CN)_6_]^4−^, one water molecule (H_2_O) of [Fe^III^(H_2_O)_6_]^3+^ is replaced by one nitrogen atom (N) of [Fe^II^(CN)_6_]^4−^, which forms the Fe^II^–CN–Fe^III^ bridging structure (Fig. 4A) [[33], [34], [35]]. In this bridging structure, the asymmetry of the cyanide ligand (–CN–) plays an important role in determining the stability of the oxidation states. The carbon atom (C) of –CN– exhibited both electron-donating and electron-accepting characteristics, stabilizing the lower oxidation state of the coordinating metal ion. On the other hand, the N atom of –CN– acted as an electron donor, and the coordinating metal ions became easy to oxidize. As a result, Fe^II^ bound to the C of –CN– remained stable in a lower oxidation state, and Fe^III^ bound to the N of –CN– remained stable in a higher oxidation state (Fig. 4B).

### 8.5 Turnbull blue

**Fig. 5:**
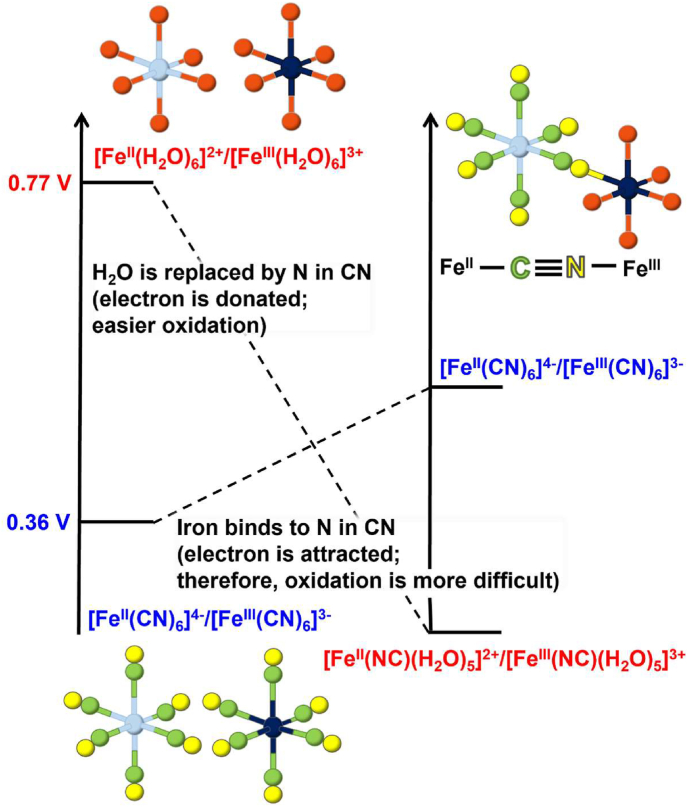
Changes in the standard electrode potential (*E*^*0*^) accompanying the formation of the Fe^II^–C–N–Fe^III^ linkage structure. Before linkage, *E*^*0*^ is +0.77 V for the Fe^II^/Fe^III^ ([Fe^II^(H_2_O)_6_]^2+^/[Fe^III^(H_2_O)_6_]^3+^) couple and +0.36 V for the [Fe^II^(CN)_6_]^4−^/[Fe^III^(CN)_6_]^3−^ couple (left). After linkage by the formation of Prussian blue or Turnbull blue, one water molecule of [Fe^II^(H_2_O)_6_]^2+^ or [Fe^III^(H_2_O)_6_]^3+^ is replaced by one N atom to give [Fe^II^(NC)(H_2_O)_5_]^2+^ or [Fe^III^(NC)(H_2_O)_5_]^3+^. The *E*^*0*^ for the resultant [Fe^II^(NC)(H_2_O)_5_]^2+^/[Fe^III^(NC)(H_2_O)_5_]^3+^ couple is lowered by several hundred millivolts. In addition, *E*^*0*^ for the [Fe^II^(CN)_6_]^4−^/[Fe^III^(CN)_6_]^3−^ couple is modestly raised after linkage, resulting in the reversion of *E*^*0*^ for these two couples (right). Because of this reversion, Fe^II^–C–N–Fe^III^ formed by the reaction between [Fe^II^(CN)_6_]^4−^ and [Fe^III^(H_2_O)_6_]^3+^ (Prussian blue) remains as it is, whereas Fe^III^–C–N–Fe^II^ formed by the reaction between [Fe^III^(CN)_6_]^3−^ and [Fe^II^(H_2_O)_6_]^2+^ (Turnbull blue) is changed to Fe^II^–C–N–Fe^III^ by inner-sphere type electron transfer.

In the formation of Turnbull blue by the reaction of [Fe^II^(H_2_O)_6_]^2+^ and [Fe^III^(CN)_6_]^3−^, one H_2_O molecule of [Fe^II^(H_2_O)_6_]^2+^ is replaced by one N of [Fe^III^(CN)_6_]^3−^ to form an Fe^III^–CN–Fe^II^ bridging structure (Fig. 4A) [[33], [34], [35]]. In this structure, the strong electron donation from N makes [Fe^II^(NC)(H_2_O)_5_]^2+^ easier to oxidize and, consequently, the redox potential is lowered by several hundred millivolts (Fig. 5). Simultaneously, the reduction potential of [Fe^III^(CN)_6_]^3−^ moiety is raised by the formation of the Fe^III^–CN–Fe^II^ bridging structure because of the electron-withdrawing effect from Fe^II^ ions (Fig. 4B). By combining these effects, an inner-sphere-type electron transfer occurs from Fe^II^ to Fe^III^ through the cyanide group. This converts Fe^III^–CN–Fe^II^ to Fe^II^–CN–Fe^III^ and yields Fe^III^[Fe^II^(CN)_6_]^−^, which is the same as Prussian blue (Fig. 4A).

## 9 Practical applications of Perls’ Prussian blue staining

In the first part of this review, we described the chemical basis of Perls' Prussian blue staining, which is not often described in the forensic literature. Following is a brief description of Perls' staining in practice: 1) the composition of staining solutions, 2) staining procedure, 3) haemosiderin and haematoidin, 4) precautions for the dating of cutaneous haemorrhage, 5) whether Perls’ staining is applicable for intracranial haemorrhage, 6) infantile pulmonary haemosiderin, 7) the detection of exogenous iron rather than endogenous iron of haemosiderin, and 8) immunohistochemistry of glycophorin A in decomposed tissues.

### 9.1 Staining solutions

Perls did not provide a formula, that is, the concentrations of K_4_[Fe^II^(CN)_6_] and HCl. Subsequent optimization resulted in two representative concentrations.

One was a mixture of the equal volumes of aqueous solutions of 2 % K_4_[Fe^II^(CN)_6_] and 2 % HCl by Bunting [37]. Potassium ferrocyanide is available as trihydrate K_4_[Fe^II^(CN)_6_]·3H_2_O and, unless otherwise stated, 2 % K_4_[Fe^II^(CN)_6_] is prepared by dissolving 2 g of the K_4_[Fe^II^(CN)_6_]·3H_2_O in 100 mL of water (H_2_O) [8,38]. Currently, this formula or modified formulae (e.g., mixtures of the same volume of 2 % K_4_[Fe^II^(CN)_6_] and 1 % HCl [38] and 1 % K_4_[Fe^II^(CN)_6_] and 2 % HCl [39]) are prevalent. Because the staining solution is not blue but thin yellow [40] and a blue colour is developed by reacting with Fe^III^(H_2_O)_6_^3+^, Perls' Prussian blue staining is also (or perhaps more appropriately) called the Perls' Prussian blue reaction (Perls' reaction). Fe^III^(H_2_O)_6_^3+^ is metastable below pH 2 and stable below pH 1 [20,32].

The other representative recipe is a mixture of equal volumes of 20 % K_4_[Fe^II^(CN)_6_] and 20 % aqueous solution of concentrated (approximately 32 % w/w) HCl by Gömöri [41]. This formulation was based on the observation that Prussian blue is soluble in water but is barely soluble in the concentrated K_4_[Fe^II^(CN)_6_] solution, preventing dissipation of the formed Prussian blue and thus allowing higher contrast staining. Another modification of this formula is to use successive incubation of tissues with HCl and then with K_4_[Fe^II^(CN)_6_] instead of using a mixture of these concentrated reagents [42].

Physicochemically, Prussian blue is soluble (dispersible) in water or insoluble depending on how it is formulated, as described above. As with Perls' staining, the resultant Prussian blue is expected to take a soluble form because K_4_[Fe^II^(CN)_6_] is present in excess compared to Fe^3+^ released from the tissues [43]. However, Prussian blue formed by Perls' staining remains on the tissue. This contradictory observation has not been well explored by histological studies, but may be explained by the rapid formation of Prussian blue at the interface between the solid phase of the tissue and the liquid phase of the staining solution. In this regard, Gömöri's observation that concentrated K_4_[Fe^II^(CN)_6_] solution prevents dissipation of the formed Prussian blue merits further investigation.

### 9.2 Staining procedure

Neutral-buffered formalin is suitable for the fixation of tissues to minimize the dissipation of iron under acidic conditions [37]. Decalcification with solutions containing formic or nitric acid reduces the amount of haemosiderin [44]. K_4_[Fe^II^(CN)_6_] in the staining solution spontaneously decomposes to release Fe^II^, which is oxidized by O_2_ and combines with ferrocyanide to form Prussian blue [37]. As decomposition proceeds, the solution changes from pale yellow to blue, and the tissues become lightly stained. Therefore, the staining solution should be prepared immediately before use, and tissues should not be immersed for more than half an hour [37].

### 9.3 Haemosiderin and haematoidin in haemorrhagic lesions

In addition to haemosiderin, haematoidin is occasionally present in traumatic lesions, particularly haematoma [15,18,21]. It is generally observed that besides residual haemosiderin of remote (old) haemorrhage, haemosiderin appears first as haemosiderin-laden macrophages, and then haematoidin appears as haematoidin-laden macrophages and/or extracellular haematoidin [16,[45], [46], [47]].

However, an essential understanding of this observation has not been advanced since the time of Perls and Neumann [21]. That is, it is not known whether the formation of haemosiderin and haematoidin occurs as a series of reactions or in parallel. In modern terms, it is not known whether the same macrophage first produces haemosiderin, then secretes accumulated haemosiderin and switches to produce haematoidin, or whether there are different lineages (subsets) of macrophages that produce and preferentially accumulate haemosiderin, haematoidin, and/or both. For these fundamental questions to be solved, many studies of various approaches will need to be conducted, including work to develop immunohistological staining approaches by forensic investigators.

### 9.4 Dating of skin wounds

There is general agreement that when dating is required for skin wounds, particularly bruises, Perls' staining can be recommended in addition to haematoxylin eosin (HE) staining. It is conventional to take tissue specimen(s) from the marginal portion of the haemorrhage because it allows for comparison with the adjacent healthy cutaneous tissue. However, in the case of bruises (blunt injuries), macrophages were found to migrate towards the damaged tissue rather than towards extravasated blood itself [48]. Thus, if only one or two specimens are taken for a single haemorrhage owing to time constraints or other reasons, the sample(s) should be taken from the central portion of the haemorrhage [49]. If multiple samplings are feasible, deeper portions involving the underlying muscle should also be taken [50].

**Fig. 6:**
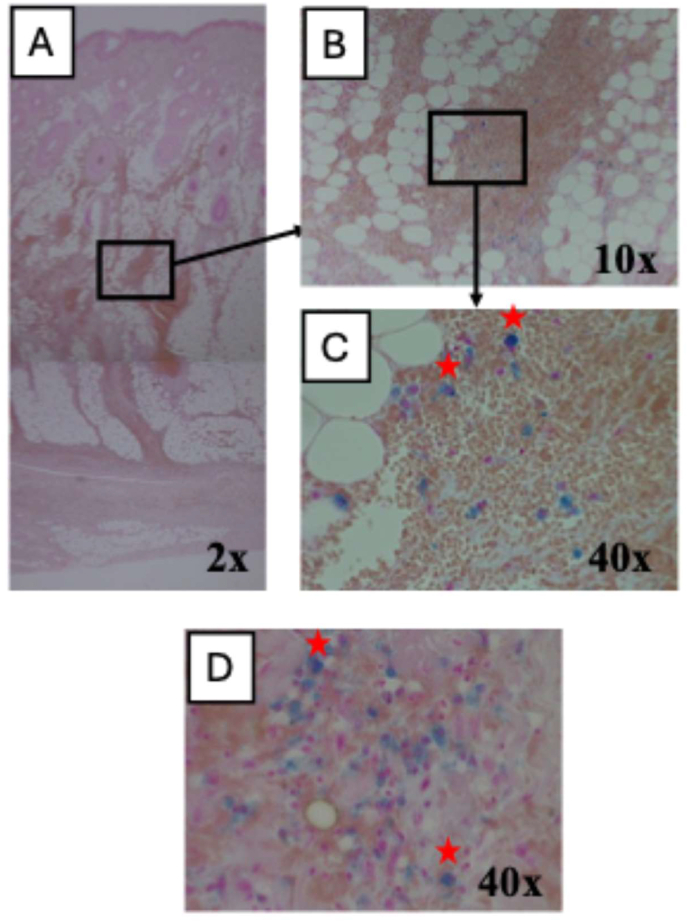
Perls' staining of the small skin haemorrhage of the head that was not noticed until reflection by scalpel at autopsy. The deceased died of acute subdural haematoma approximately one day after presenting to a hospital with vomiting. (A) Microscopic image of entire skin (2x). (B) Magnification (10×) of a portion in subcutaneous adipose tissue (layer between dermis and epicranial apponeurosis). Haemosiderin is observed as blue spots. (C) Further magnification (40×). The small spots were identified as haemosiderin-laden macrophages. (D) Haemosiderin-laden macrophages in a region not shown in A under the same magnification (40×).

Regarding the chronology of skin wounds, Knight's Forensic Pathology [2] provides the following description: “Haemosiderin become stainable from about the third day onwards, if there is a bruise or any bleeding into the wound, but is often not obtainable by Perls' reaction until the fifth day (four days after the injury) even though some claim it can appear on the first or second day.” Vanezis [1] states, “Haemosiderin-laden macrophages have been seen in the skin and subcutaneous tissue as early as 24–48 h after the infliction of trauma and more commonly from 4 to 8 days.” We have relied on these descriptions to date skin wounds, as seems to be the consensus of many forensic scientists. Fig. 6A shows Perls' staining of a small subcutaneous haemorrhage of the head in the deceased who presented with vomiting to a hospital and died of a subdural haematoma the next day. At 4× magnification, blue spots scattered with a moderate density were easily observed, but 10× objective magnification was required to record an image (Fig. 6B). At higher magnification of 40× (Fig. 6C and D), the blue spots were identified as haemosiderin-laden macrophages. From the results of Perls' staining, the bleeding was estimated to have occurred approximately three or four days prior.

Caution should be paid to bruises in infants and young children because neutrophil infiltration and subsequent macrophage infiltration can be considerably delayed [51].

### 9.5 Limitations in dating intracranial haemorrhages and child ocular and retroocular haemorrhages

When dating intracranial haemorrhage, particularly subdural haemorrhage/haematoma, is required, Perls' staining is recommended in addition to HE staining, as in the case of skin wounds [2,52]. However, care should be taken in interpreting the results, with particular attention paid to observations that chronological changes in infants would differ from adults [53] and that residual haemosiderin-laden macrophages are common in paediatric dura mater [54]. Perls' staining has also been used to evaluate haemorrhage in the ocular regions of children with suspected abusive head trauma (AHT) [55,56].

Serious arguments that Perls' staining is unnecessary or even misleading have been raised for intracranial haemorrhages by Castellani et al. [42] and for paediatric retroocular and subdural haemorrhages by Del Bigio and Phillis [57]. These studies are convincing, and forensic practitioners who have been performing Perls' staining for such haemorrhages are advised to consider whether to continue Perls' staining, narrow the selection of cases examined by staining, or abandon staining. In addition, the article by Del Bigio and Phillis [57] and the review on brain trauma by Rahaman and Del Bigio [15] are quite informative regarding the degradation of haemoglobin to haemosiderin and the recommendation for consultation when performing Perls' staining for the dating of skin bleeds as well as intracranial haemorrhages.

### 9.6 Infantile pulmonary haemosiderin

Pulmonary haemosiderin-laden macrophages in infants were, together with alveolar haemorrhage, once associated with fatal or previous episodes of upper airway obstruction, but after thorough evaluation neither are considered as diagnostic of such obstructions [58,59]. While significant alveolar haemorrhage leaves haemosiderin-laden macrophages, they are observed in many other pulmonary or extra-pulmonary diseases [60].

### 9.7 Exogenous iron and asbestos

Iron is rarely transferred from a firearm to the grasping hand, which is called "rust stain" [61]. This phenomenon resembles "rusters" that readily corrode metal in the precision engineering industry, as the mechanism is oxidation of iron in the presence of chloride ions (Cl^−^) in sweat (Fe → Fe^2+^ + 2e^−^) [62]. The released Fe^II^ is oxidized by O_2_ to Fe^III^, which is detected by Perls’ staining [61].

Ingestion of a button battery (1.5 or 3 V) is fatal unless it is quickly removed [63]. If the battery becomes lodged in a narrow portion of the oesophagus where water is electrolysed (reduced) at the negative pole to produce molecular hydrogen (H_2_) and hydroxide (OH^−^), the OH^−^ causes serious injury to the oesophagus and surrounding structures. At the positive pole, iron constituting the metal case is thought to be oxidized and released as Fe^II^ in the same way as the rust stain [64]. The Fe^II^ permeates to the tissues, and under alkali condition, these divalent species are easily oxidized by O_2_, which can be detected by Perls' staining [63].

Many types of asbestos fibres contain iron, and meticulous sampling of lung tissue and Perls' staining of thick tissues sections (30 μm) are required for the diagnosis of asbestosis [65]. Although some types do not contain iron, endogenous iron that is adsorbed on the fibres during insidious inflammation does stain blue [60].

### 9.8 Perls’ staining to complement immunohistochemical detection of glycophorin A in decomposed tissues

Returning to endogenous haemosiderin, the presence of extravasated red blood cells adjacent to haemosiderin-laden macrophages, as shown in Fig. 6, is crucial for estimating the time since bleeding because it excludes or at least decreases the possibility that haemosiderin-laden macrophages are incidental residual ones that persist from remote (old) haemorrhages irrelevant to death. However, as the decomposition of bodies advances, the shape of the red blood cells becomes blurred and eventually the cells disappear, rendering microscopic identification of haemorrhages by HE staining impossible [66,67]. In addition, haemoglobin easily diffuses out of the blood vessels after death [68], making differentiation of haemorrhages from postmortem lividity difficult [2,69,70].

On the other hand, experts in forensic pathology recommend immunohistochemical detection of glycophorin A as a reliable marker for the presence of red blood cells [66,67,[70], [71], [72]]. Glycophorin A is a major transmembranous sialoglycoprotein of red blood cells [[71], [72], [73]]. It exists as a dimer and its monomer consists of 131 amino acids (polymorphism manifested as the MN blood group) and 16 oligosaccharides, accounting for one-third of the molecule [73,74]. Notably, in contrast to haemoglobin, glycophorin A was found to remain within the blood vessels after death [[75], [76], [77]]. Thus, the presence of glycophorin A outside of blood vessels or in areas devoid of vessels indicates extravasation of red blood cells (haemorrhages) [[70], [71], [72]]. In addition, glycophorin A resists postmortem degradation, allowing the identification of haemorrhage in decomposed tissues [67]. However, extravasation of red blood cells can occur after death [78,79], and antemortem (true) haemorrhages cannot be differentiated from such postmortem haemorrhages by glycophorin A alone.

Based on these considerations, Perls’ staining is expected to complement the immunohistochemical detection of glycophorin A. Indeed, Di Fazio et al. [80] detected glycophorin A in the encephalic structure and haemosiderin in the brain stem of a homicide victim who had died of chronic subdural haematoma and was exhumed and autopsied as many as 742 days after death [72], demonstrating the reinforcement of glycophorin A and haemosiderin findings to confirm the existence of haemorrhages of several days.

## 10 Concluding remarks

Perls' staining or reaction is used to detect haemosiderin that is stored or sequestrated non-haeme iron in tissues. Methodologically, Fe^3+^ (Fe^III^) is released by HCl and reacts with K_4_[Fe^II^(CN)_6_] to form Prussian blue. The resulting structure is a cubic lattice with Fe^II^ or Fe^III^ ions alternately aligned at the corners to give Fe^II^–CN–Fe^III^ formations at the edges. Turnbull blue is formed by the reaction between Fe^2+^ (Fe^II^) and K_3_[Fe^III^(CN)_6_] and through an inner-sphere electron transfer from Fe^II^ to Fe^III^, resulting in a structure that is the same as that of Prussian blue.

In tissues and organs where haemosiderin is not usually present, or in traumatized areas, its presence is of forensic significance. Most commonly, bruising (blunt cutaneous injuries) and subdural haemorrhages/haematomas are examined in practice, and the detection of haemosiderin-laden macrophages (siderophages) has been used as a marker for the estimation of time since bleeding. Background staining is minimal because haeme iron is not released by HCl and because iron released from ferritin, another storage non-haeme iron, is usually scarce for microscopic detection. Thus, the detection of haemosiderin-laden macrophages by microscopy is easy, increasing the usefulness of Perls’ staining. However, its reliability in intracranial and ocular regions is controversial. In addition, in organs such as the liver where iron metabolism is active, ferritin can occasionally be seen as a light blue blush when accumulated in the constituent cells [81].

Finally, the reason why Prussian blue is blue is described. In general, the colour of transition metal compounds is based on electron transitions within the same metal ion consequent upon the absorption of light. However, the intense blue colour of Prussian blue (Turnbull blue) is due to the intervalence charge transfer between Fe^II^ and Fe^III^ with significant electronic interactions through the bridging cyanide group (Fe^II^–CN–Fe^III^) [6,36]. The peak absorption of light is about 700 nm (orange), and hence the light reflected from Prussian blue appears blue [82].

## CRediT authorship contribution statement

**Ai Sonoda:** Writing – original draft. **Masayuki Nihei:** Writing – review & editing. **Norihiro Shinkawa:** Supervision. **Eiji Kakizaki:** Writing – review & editing. **Nobuhiro Yukawa:** Writing – review & editing.

## Funding details and disclosure statement

This research did not receive any specific grants from funding agencies in the public, commercial, or not-for-profit sectors, there is no conflict of interest.

## Declaration of competing interest

Conflicts of Interest and Source of Funding: This research did not receive any specific grants from funding agencies in the public, commercial, or not-for-profit sectors, there is no conflict of interest.

## References

[1] Vanezis P., Rutty N.G. (2001). Essentials of Autopsy Practice.

[2] Saukko P., Knight B. (2016).

[3] Roth K. (2021). Berliner Blau - entdeckner und Verräter. Chem. Unserer Zeit.

[4] Grohe F. (1886). Beiträge zur Pathologischen Anatomie und Physiologie. I. Zur Geschichte der Melanämie nebst Bemerkungen über den normalen Bau der Milz und Lymphdrüse. Virchows Arch. Pathol. Anat..

[5] Perls M. (1867). Nachweis von Eisenoxyd in gewissen Pigmenten. Arch. für Pathol. Anat. Physiol. für Klin. Med..

[6] Ware M. (2008). Prussian blue: artists' pigment and chemists' sponge. J. Chem. Educ..

[7] Kraft A. (2021). Some considerations on the structure, composition, and properties of Prussian blue: a contribution to the current discussion. Ionics.

[8] Lillie R.D., Geer J.C. (1965). On the relation of enterosiderosis pigments of man and Guinea pig. Melanosis and pseudomelanosis of Colon and villi and the intestinal iron uptake and storage mechanism. Am. J. Pathol..

[9] Meguro R., Asano Y., Odagiri S., Li C., Iwatsuki H., Shoumura K. (2007). Nonheme- iron histochemistry for light and electron microscopy: a historical, theoretical and technical review. Arch. Histol. Cytol..

[10] Klages U., Wilhelmi F. (1974).

[11] Mori T., Nagata K., Town T., Tan J., Matsui T., Asano T. (2001). Intracisternal increase of superoxide anion production in a canine subarachnoid hemorrhage model. Stroke.

[12] Riße M., Weiler G. (1987). Hämosiderinbefunde in Leber, Milz und Lunge bei Neugeborenen und Säuglingen. Z. Rechtsmed..

[13] Takahashi S., Takada A., Saito K., Hara M., Yoneyama K., Nakanishi H. (2022). Diagnostic significance of the histopathology of bone marrow macrophages in forensic autopsies. Leg. Med..

[14] Saito H. (2014). Metabolism of iron stores. Nagoya J. Med. Sci..

[15] Rahaman P., Del Bigio M.R. (2018). Histology of brain trauma and hypoxia-ischemia. Acad. Forensic Pathol..

[16] Betz P. (1994). Histological and enzyme histochemical parameters for the age estimation of human skin wounds. Int. J. Leg. Med..

[17] Langlois N.E.I., Olds K., Ross C., Byard R.W. (2015). Heme oxygenase-1 and heme oxygenase-2 expression in bruises, forensic sci. Med. Pathol..

[18] Muir R., Niven J.S. (1935). The local formation of blood pigments. J. Pathol..

[19] Macaluum A.B. (1895). On the distribution of assimilated iron from compounds, other than haemoglobin and haematins, in animal and vegetable cells. J. Cell Sci..

[20] Curdt F., Haase K., Ziegenbalg L., Greb H., Heyers D., Winklhofer M. (2022). Prussian blue technique is prone to yield false negative results in magnetoreception research. Sci. Rep..

[21] Neumann E. (1888). Beiträge zur Kenntniss der pathologischen Pigmente. Arch. für Pathol. Anat. Physiol. für Klin. Med..

[22] Behera R.K., Torres R., Tosha T., Bradley J.M., Goulding C.W., Elizabeth C., Theil E.C. (2015). Fe^2+^ substrate transport through ferritin protein cage ion channels influences enzyme activity and biomineralization. J. Biol. Inorg. Chem..

[23] van Dujin S., Nabuurs R.J.A., van Duinen S.G., Natte R. (2013). Comparison of histological techniques to visualize iron in paraffin-embedded brain tissue of patients with alzheimer's disease. J. Histochem. Cytochem..

[24] Richter G.W. (1978). The iron-loaded cell – the cytopathology of iron storage. Am. J. Pathol..

[25] Wixom R.L., Prutkin L., Munro H.N. (1980). Hemosiderin: nature, formation, and significance. Int. Rev. Exp. Pathol..

[26] Koeppen A.H. (1995). The history of iron in brain. J. Neurol. Sci..

[27] Shinkawa N., Takahashi N., Yano K., Sawaguchi A., Sonoda A., Kakizaki E., Yukawa N. (2023). A suggested mechanism for green coloration of the postmortem brain. Am. J. Forensic Med. Pathol.

[28] Larson C.P., Reberger C.C., Wicks M.J. (1964). Unusual cases. 2 – the purple brain death. Med. Sci. Law.

[29] Shinkawa N., Sonoda A., Kakizaki E., Yukawa N. (2023). Hemorrhagic shock due to ruptured idiopathic intramural hematoma of the sigmoid colon – an autopsy case report. Radiol. Case Rep..

[30] Hayashi E. (2011). Teaching materials for visualization of oxidation-reduction reactions using Prussian blue. https://www.toray-sf.or.jp/awards/education/pdf/h22_06.pdf.

[31] Hansen L.D., Litman W.M., Daub G. (1969). Turnbull's blue and Prussian blue: KFe(III)[Fe(II)(CN)_6_]. J. Chem. Educ..

[32] Koppenol W.H., Hider R.H. (2019). Iron and redox cycling. Do's and don'ts. Free Radic. Biol. Med..

[33] Taube H. (1984). Electron transfer between metal complexes – a retrospective view (Nobel Lecture). Angew Chem. Int. Ed. Engl..

[34] Bernhardt P.V., Bozoglían F., Macpherson B.P., Martínez M. (2005). Molecular mixed-valence cyanide bridged Co^3+^–Fe^2+^ complexes. Coord. Chem. Rev..

[35] Cafun J.D., Champion G., Arrio M.A., dit Moulin C.C., Bleuzen A. (2010). Photomagnetic CoFe Prussian blue analogues: role of the cyanide ions as active electron transfer bridges modulated by cyanide-alkali metal ion interactions. J. Am. Chem. Soc..

[36] Nihei M. (2020). Molecular Prussian blue analogues: from bulk to molecules and low- dimensional aggregates. Chem. Lett..

[37] Bunting H. (1949). The histochemical detection of iron in tissues. Stain Technol..

[38] Watanabe A., Mizuguchi K. (2011). Medical Technology Supplement. all About Staining Methods.

[39] Orchard G.E., Suvarna S.K., Layton C., Bancroft J.D. (2019). Bancroft's Theory and Practice of Histological Techniques.

[40] Musshoff F., Kirschbaum K.M., Madea B. (2011). An uncommon case of a suicide with inhalation of hydrogen cyanide. Forensic Sci. Int..

[41] Gömöri G. (1936). Microtechnical demonstration of iron. A criticism of its methods. Am. J. Pathol..

[42] Castellani R.J., Mojica G., Perry G. (2016). The role of the iron stain in assessing intracranial hemorrhage. Open Neurol. J..

[43] Hirose S., Yasutomi M., Murai N., Yamada R., Katayama A., Tsujino M., Iwasa Z., Razzaq A.K.A. (1970). A modified method of the Prussian blue reaction for the histochemical demonstration of iron, and its application to the colloidal iron reaction of acid mucopolysaccharides. Acta Histochem. Cytoc..

[44] Byard R.W., Bellis M. (2010). The effect of decalcifying solutions on hemosiderin staining. J. Forensic Sci..

[45] Laiho K., Oehmichen M M., Kirchner H. (1995). The Wound Healing Process: Forensic Pathological Aspects.

[46] Walter T., Meissner C., Oehmichen M. (2009). Pathomorphological staging of subdural hemorrhages: statistical analysis of posttraumatic histomorphological alterations. Leg. Med..

[47] van den Bos D., Zomer S., Kubat B. (2014). Dare to date: age estimation of subdural hematomas, literature, and case analysis. Int. J. Leg. Med..

[48] Ross C., Byard R.W. (2013). Does the intensity of the inflammatory reaction in a bruise depend on its proximity to the site of trauma?. Forensic Sci. Med. Pathol..

[49] Ross C., Langlois N.E.I., Heath K., Byard R.W. (2015). Further evidence for a lack of reliability in the histologic ageing of bruises – an autopsy study. Aust. J. Forensic Sci..

[50] Maggioni L., Maderna E., Gorio M.C., Cappella A., Andreola S., Bulfamante G., Cattaneo C. (2021). The frequently dismissed importance of properly sampling skin bruises. Leg. Med..

[51] Byard R.W., Wick R., Gilbert J.D., Donald T. (2008). Histologic dating of bruises in moribund infants and young children. Forensic Sci. Med. Pathol..

[52] Oehmichen M., Auer R.N., König H.G. (2009).

[53] Delteil C., Humez S., Boucekine M., Jouvet A., Hedouin V., Fanton L., Leonetti G., Tuchtan L., Piercecchi M.-D. (2019). Histological dating of subdural hematoma in infants. Int. J. Leg. Med..

[54] Kywanczyk A., Bundock E.A. (2018). Quantifying macrophages and haemosiderin in pediatric dura mater. J. Forensic Sci..

[55] Delteil C., Kolopp M., Capuani C., Humez S., Boucekine M., Leonetti G., Torrents J., Tuchtan L., Piercecchi M.-D. (2019). Histological dating of subarachnoid hemorrhage and retinal hemorrhage in infants. Forensic Sci. Int..

[56] Di Fazio N., Delogu G., Morena D., Cipolloni L., Scopetti M., Mazzilli S., Frati P., Fineschi V. (2023). New insights into the diagnosis and age distribution of retinal hemorrhages from abusive head trauma: a systemic review. Diagnostics.

[57] Del Bigio M.R., Phillips S.M. (2017). Retroocular and subdural hemorrhage or hemosiderin deposits in pediatric autopsies. J. Neuropathol. Exp. Neurol..

[58] Forbes A., Acland P. (2004). What is the significance of haemosiderin in the lungs of deceased infants?. Med. Sci. Law.

[59] Kepron C., Walker A., Milroy C.A. (2016). Are there hallmarks of child abuse? II. Non-osseous injuries. Acad. Forensic Pathol..

[60] Ghio A.J., Roggli V.L. (2021). Perls' Prussian blue stains of lung tissues, bronchioalveolar lavage, and sputum. J. Environ. Pathol. Toxicol. Oncol..

[61] Tomassini L., Paolini D., Manta A.M., Bottni E., Ciallella C. (2021). "Rust stain": a rare mark in firearm suicide - a case report and review of the literature. Int. J. Leg. Med..

[62] Burton J.L., Pye R.J., Brookes D.B. (1976). Metal corrosion by chloride in sweat. The problem of 'rusters' in industry. Br. J. Dermatol..

[63] LaFrance D.R., Traylor J.G., Jin L. (2011). Aspiration pneumonia and esophageal fistula secondary to button battery ingestion. Forensic Sci. Med. Pathol..

[64] Shinkawa N., Meiri T., Kakizaki E., Sonoda A., Yukawa N. (2021). “Black ring-shaped burn” in button battery ingestion is not a burn – Comparison with charring using spectral CT. Br. J. Radiol..

[65] Cirielli V., Bortolotti F., Cima L., De Battisti Z., Del Balzo G., De Salvia A., Laposata C., Raniero D., Vermiglio E., Portas M., Rodegher P., Ghimenton C., Martignoni G., Eccher A., Narayanasamy M., Vergine M., Turrina S., Tagliaro F., De Leo D., Brunelli M. (2021). Consultation between forensic and clinical pathologists for histopathology examination after forensic autopsy. Med. Sci. Law.

[66] Baldari B., Vittorio S., Sessa F., Cipolloni L., Bertozzi G., Neri M., Cantatore S., Fineschi V., Aromatario M. (2021). Forensic application of monoclonal anti-human. glycophorin A antibody in samples from decomposed bodies to establish vitality of the injuries. A preliminary experimental study. Healthcare (Basel).

[67] Bertozzi G., Ferrara M., La Russa R., Pollice G., Gurgoglione G., Frisoni P., Alfieri L., De Simone S., Neri M., Cipolloni L. (2021). Wound vitality in decomposed. bodies: new frontiers through immunohistochemistry. Front. Med..

[68] Skopp G., Pötsch L., Lutz R., Ganssmann B., Mattern R. (1998). Hemoglobin diffusion across a venous wall: an experimental study. Am. J. Forensic Med. Pathol.

[69] Tabata N. (1995). Immunohistochemical studies on postmortem lividity. Forensic Sci. Int..

[70] Vignali G., Franceschetti L., Attisano G.C.L., Cattaneo C. (2023). Assessing wound. vitality in decomposed bodies: a review of the literature. Int. J. Leg. Med..

[71] Salzillo C., Innamorato L., Leggio A., Marzullo A. (2024). Immunohistochemical markers in the determination of lesion viability in decomposed bodies: a mini. literature review. Forensic Sci. Int..

[72] Morena D., Manta A.M., Santurro A., Scopetti M., Turillazzi E., Fineschi V. (2025). Bloody evidence: the validity of glycophorin A in the determination of wound. vitality−A systematic review of the literature. Int. J. Mol. Sci..

[73] Aoki T. (2017). A comprehensive review of our current understanding of red blood cell. (RBC) glycoproteins. Membranes.

[74] Tomita M., Furthmayr H., Marchesi V.T. (1978). Primary structure of human erythrocyte glycophorin A. Isolation and characterization of peptides and. complete amino acid sequence. Biochemistry.

[75] Kibayashi K., Higashi T., Tsunenari S. (1991). A differential study between antemortem bleeding and a postmortem infiltration of hemoglobin. Nihon Hoigaku Zasshi.

[76] Kibayashi K., Hamada K., Honjyo K., Tsunenari S. (1993). Differentiation between. bruises and putrefactive discolorations of the skin by immunological analysis of glycophorin A. Forensic Sci. Int..

[77] Tabata T., Morita M. (1997). Immunohistochemical demonstration of bleeding in decomposed bodies by using anti-glycophorin A monoclonal antibody, forensic. Sci. Int..

[78] Rutty G.N., Rutty G.N. (2001).

[79] Pollanen M.S., Perea C., Clutterbuck D.J. (2009). Hemorrhagic lividity of the neck: controlled induction of postmortem hypostatic hemorrhages. Am. J. Forensic Med. Pathol.

[80] Di Fazio N., Scopetti M., Delogu G., Morena D., Santurro A., Cipolloni L., Serviddio G., Papi L., Frati P., Turillazzi E., Fineschi V. (2023). Fourteen deaths from suspected heparin overdose in an Italian primary-level hospital. Diagnostics.

[81] Torbenson M. (2011). Iron in the liver: a review for surgical pathologists. Adv. Anat. Pathol..

[82] Dacarro G., Taglietti A., Pallavicini P. (2018). Prussian blue nanoparticles as a versatile photothermal tool. Molecules.

